# Expanding the scope of rhomboid flap: Large cutaneous defect reconstruction. Case report

**DOI:** 10.1016/j.amsu.2021.01.082

**Published:** 2021-01-26

**Authors:** Ajaipal S. Kang, Kevin S. Kang

**Affiliations:** a Department of Surgery and Chief of Plastic Surgery, UPMC Hamot, Erie, PA, 16507, USA; b Geisel Dartmouth Medical School, Hanover, NH, 03755, USA

**Keywords:** Rhomboid flap, Transposition flap, Limberg flap, Skin cancer, Skin defect

## Abstract

**Introduction:**

and importance: Large cutaneous defects may result from excision of skin malignancies. Typically, skin grafting is used to manage such defects, but the final result may be compromised by inadequate take and poor cosmesis. Accordingly, transposition flaps may be indicated.

Case Presentation and clinical discussion: A 93-year-old female presented with a painful, necrotic 12 cm × 12 cm Squamous Cell Cancer of left upper back. She underwent wide excision followed by a rhomboid transposition fasciocutaneous flap. The flap was easily designed, quickly executed, and did not require any special instruments. The overall result was a good cosmetic outcome with no complications.

**Conclusion:**

Our case outlines successful use of rhomboid flap instead of a more complicated option to reconstruct a very large cutaneous defect. The flap healed with excellent contour, texture, thickness, and color match.

## Highlights

• A very large cutaneous defect was caused by excision of a Squamous Cell Cancer in an elderly female.
• A rhomboid fasciocutaneous flap was easily designed, quickly executed, and inset without any special instruments.
• The patient healed uneventfully with excellent contour, texture, thickness, and color match.
• The excision design, flap elevation, rotation, inset, and post-operative photographs are included to educate readers.
• The case has been presented based on SCARE guidelines.

## 1 Introduction

The concept of the “reconstructive ladder” has origins in ancient Egyptian medical texts that were written sometime between 2600 and 2200 BCE. The principle suggests that simplest effective technique should be considered first in reconstruction. It was not until between 25 BCE and 50 CE, that advancements flaps began to be used in Rome [1].

Rhomboid flap was first described by Alexander Alexandrovich Limberg in 1928. The traditional design consists of a parallelogram with 2 angles of 120° and 2 angles of 60°. This transpositional flap design consists of skin and subcutaneous tissue rotated around a pivot point into an adjacent defect [2,3]. This full-thickness cutaneous local flaps typically relies on dermal–subdermal plexus blood supply [2,4]. The rhomboid flap is popular and can be used to reconstruct defects in most parts of the body. Over the years, several modifications have been reported [2,5]. Traditionally, rhomboid flaps have been safely used to reconstruct small to moderately sized skin defects [6].

This report presents a case where a large 20 cm × 15 cm defect was successfully reconstructed with a rhomboid flap. This case has been reported in line with the SCARE criteria [7].

## 2 Presentation of case

**Fig. 1:**
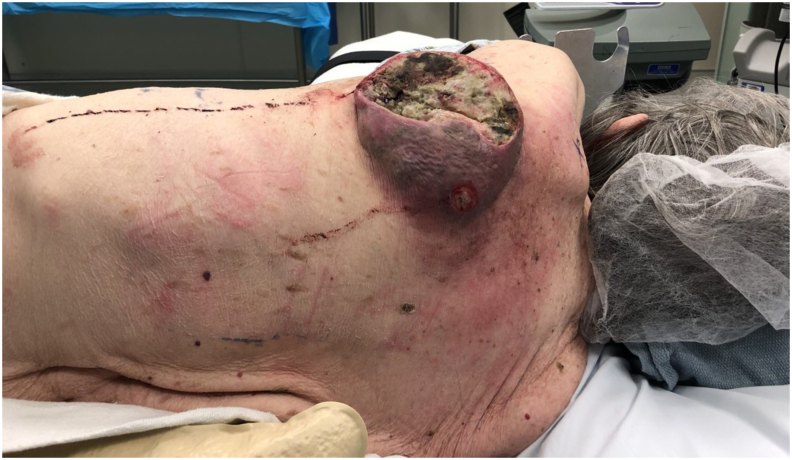
Preoperative view of left upper back Squamous Cell Cancer. Patient is in right lateral decubitus position. The head is on the right of the photograph.

A 93-year-old Caucasian healthy female with considerable sun exposure history, presented initially to her primary care physician, with a several-year history of a nonhealing ulcerated mass of left upper back (Fig. 1). The primary physician sent her to the emergency department via private vehicle. After initial evaluation, emergency department referred the patient to my clinic. She stated that the mass had become progressively larger, ulcerated, and painful. She reported a previous personal history for a Basal Cell skin cancer. Her family history was also positive for skin cancer. On physical examination, she was a Fitzpatrick class 2 skin type, elderly healthy woman with a body mass index of 20.8 kg/m3. The examination of the left upper back skin revealed a 12.0 cm × 12.0 cm diameter ulcerated, necrotic, malodorous, minimally pigmented mass lesion, tender to palpation and with mild drainage. The presumed diagnosis was a cutaneous malignancy and decision was made to proceed with wide excision and reconstruction.

**Fig. 2:**
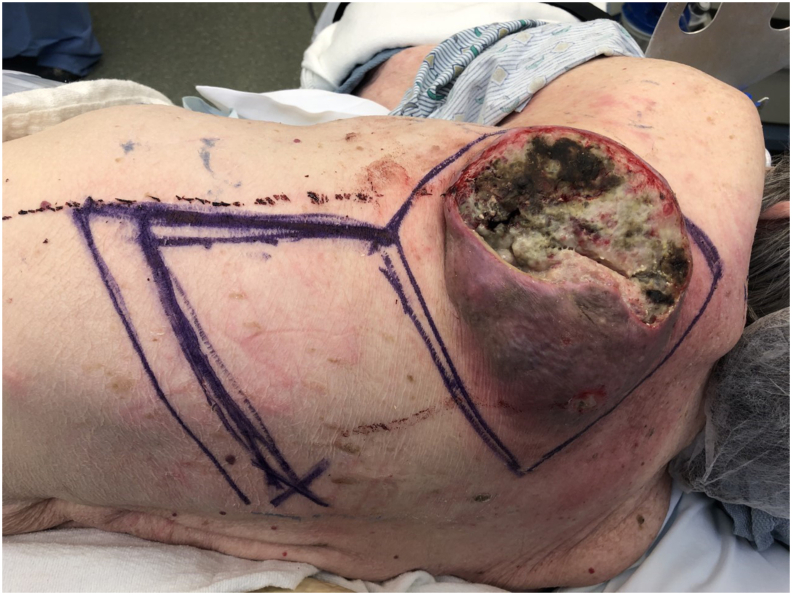
Preoperative view of left upper back Squamous Cell Cancer showing the marking (in blue) for rhomboid flap. X represents the location of perforator. Patient is in right lateral decubitus position. The head is on the right of the photograph. (For interpretation of the references to color in this figure legend, the reader is referred to the Web version of this article.)

**Fig. 3:**
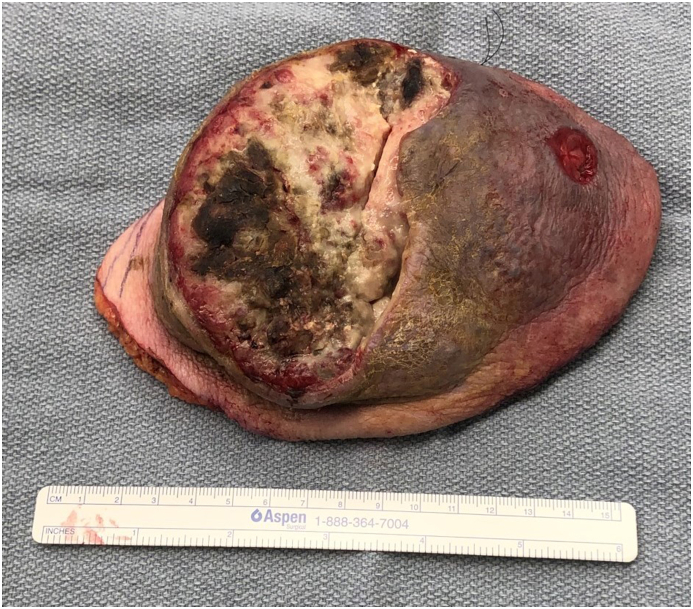
Intraoperative photograph of the excised Squamous Cell Cancer of left upper back. The scale represents 15-cm span.

**Fig. 4:**
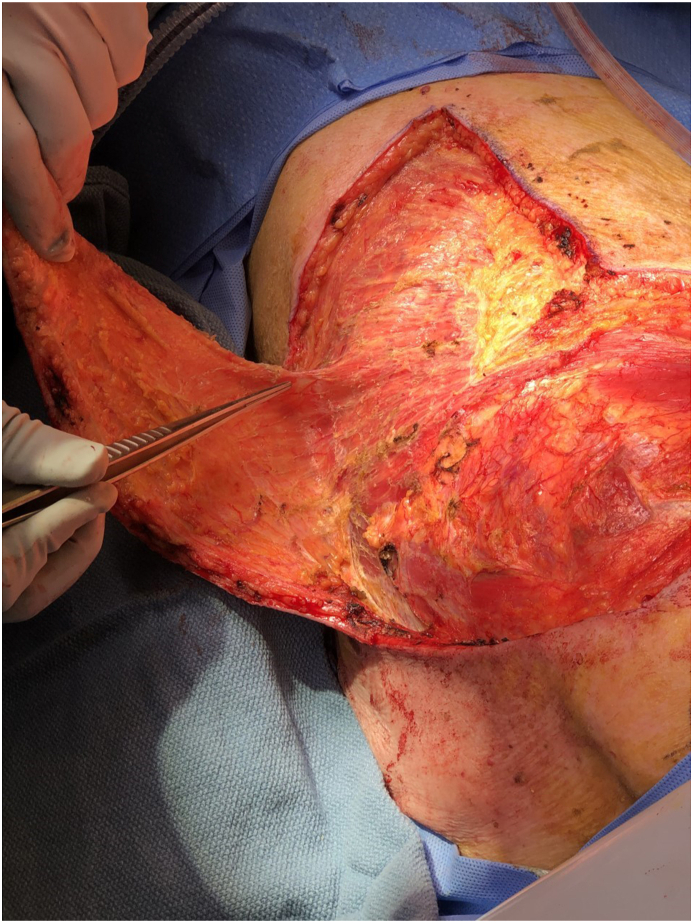
Intraoperative view of elevation of rhomboid flap for reconstruction. The tip of forceps demonstrates the location of perforator. Patient is in right lateral decubitus position. The head is at the bottom and feet at the top of the photograph.

**Fig. 5:**
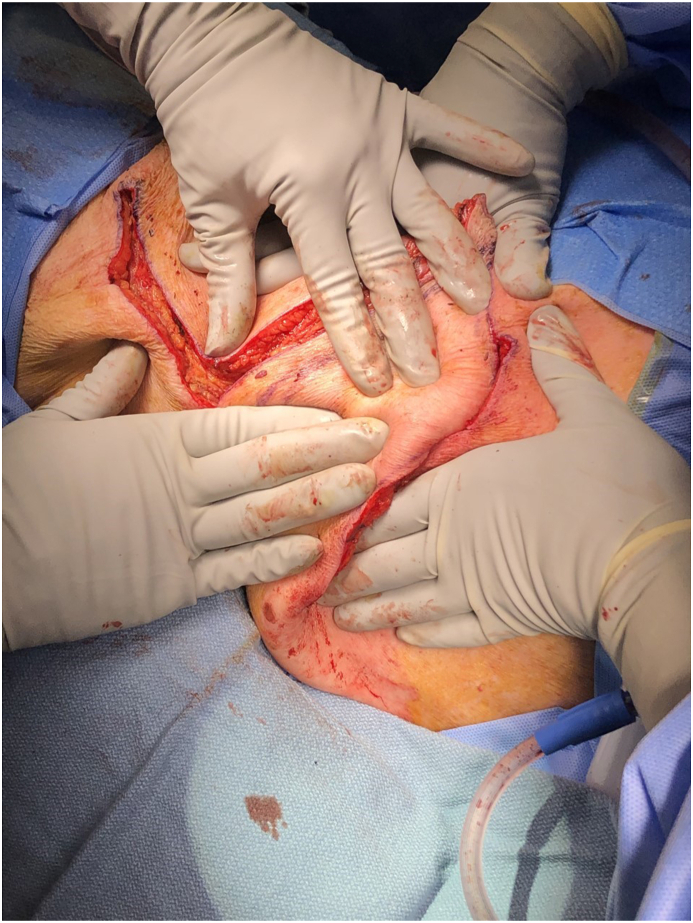
Intraoperative view of superior and lateral rotation/advancement of rhomboid flap for reconstruction. Patient is in right lateral decubitus position. The head is at the bottom and feet at the top of the photograph.

**Fig. 6:**
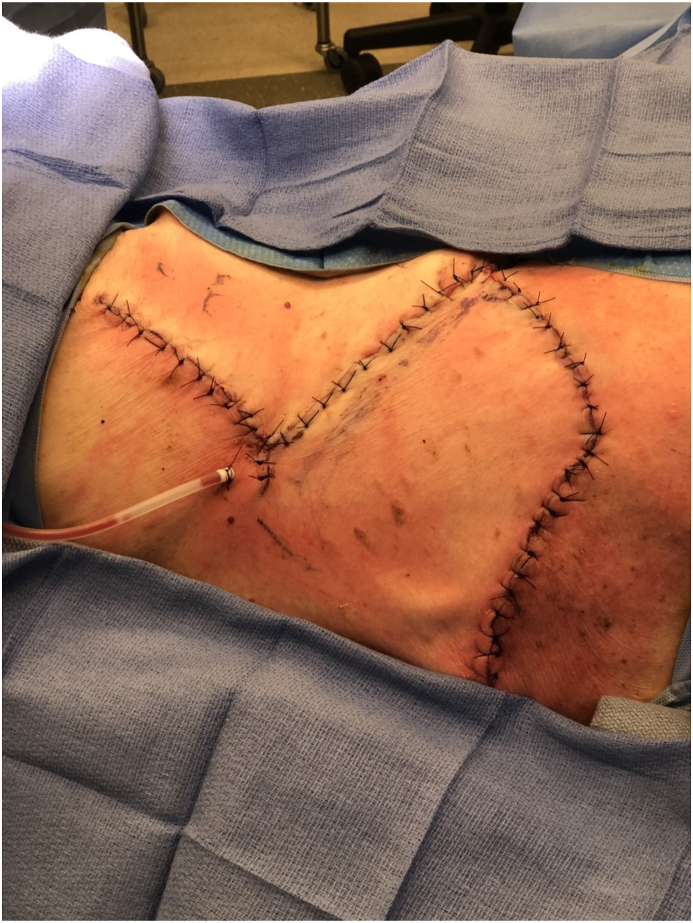
Intraoperative view of inset of rhomboid flap for reconstruction. Patient is in right lateral decubitus position. The head is on the right of the photograph.

Under general anesthesia, a 20 cm × 15 cm large necrotic mass with margins was marked to be excised. A rhomboid flap was marked along the inferior aspect of the defect because of the maximum skin laxity and resting skin tension lines. A doppler ultrasound was used to identify perforators in the territories of parascapular artery and thoracodorsal artery (Fig. 2). A 20 cm × 15 cm mass was excised full thickness all the way down to the muscle and submitted to pathology (Fig. 3). On frozen section pathology, this was an invasive moderate differentiated squamous carcinoma with negative margins. A large defect was left in place and a rhomboid flap was raised in a fasciocutaneous plane at the level of underlying muscle (Fig. 4). The flap was rotated superiorly and laterally to obliterate the defect (Fig. 5) and inset using numerous 2–0 Polysorb interrupted sutures and 2-0 Nylon sutures (Fig. 6). Both primary and secondary defects were obliterated under acceptable tension.

**Fig. 7:**
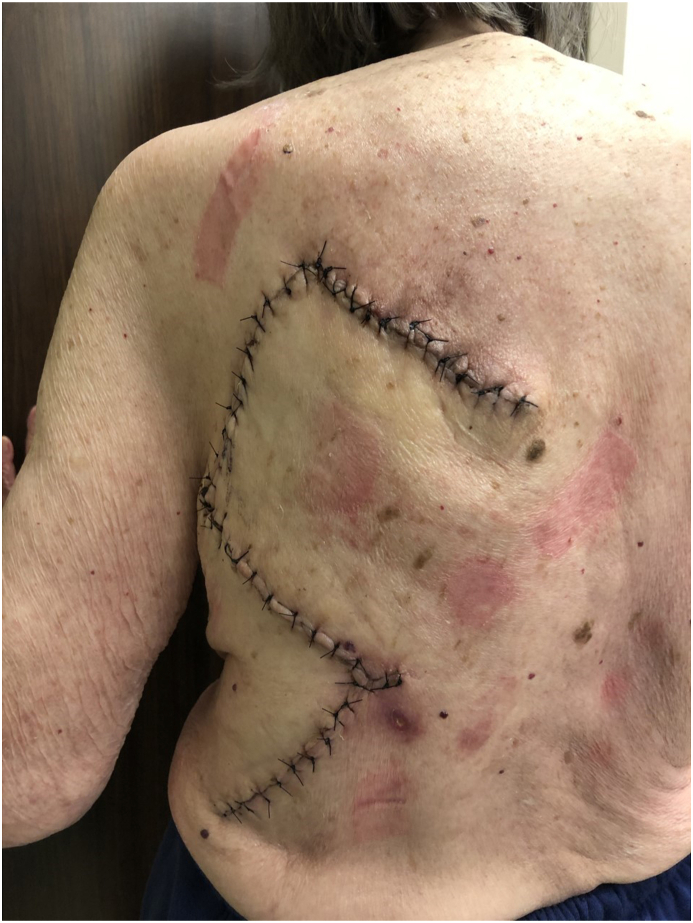
1-Month postoperative view of completely viable rhomboid flap reconstruction of cutaneous defect. Patient is standing with face away from the camera.

In the immediate postoperative period, the patient was asked to limit left shoulder mobility. The maximum tension was at pivot point. The healing was completely uneventful. Given her age, the patient elected to decline any further oncologic treatment. At 1-month follow-up, the flap healed completely without problems or limitations (Fig. 7).

## 3 Discussion

Plastic surgery is a unique specialty where numerous acceptable options may exist for management of a single defect [6]. Each reconstruction should be tailored to the unique characteristics of the defect, patient expectations, and surgeon's experience [8]. The reconstructive ladder framework suggests using first the simplest technique that proves effective [1,8]. At times, primary closure and skin grafts may lead to distortion, contour deformity, or unacceptable scarring. Such instances, even for small lesions, are more suitable for skin flaps [6,9].

An elliptical excision with primary closure may leave a central depression, flat contour and “dog ear” peaks on both corners [5,10]. To avoid this deformity, an incision length-to-width ratio of 3:1 is required, creating a longer linear scar [10,11]. Unfortunately, larger portions of healthy skin around the defect are sacrificed, and aesthetic outcomes maybe compromised [10,11]. These issues are even more magnified in areas of greater tension, such as proximity to a joint [5]. Well designed local flaps avoid these limitations [6].

Rhomboid flap design is chosen such that line of donor closure is placed along the line of maximal extensibility closure resulting in better distribution of tension. The participation of surrounding skin also reduces tension [5,6]. Less tension means improved chances of healing and less risk of distortion of adjacent anatomic architecture [6]. The “broken” geometric final scar appearance also makes it less noticeable [6]. A recent meta-analysis comparison with primary closure, for sacrococcygeal pilonidal surgeries, showed rhomboid flaps resulted in a lower relative risk of dehiscence and wound infection [12].

The presented case further highlights the versatility of the rhomboid flap design. The 20 cm × 15 cm-sized cutaneous defect was successfully reconstructed. Unlike the typical purely random pattern flap, this was a fasciocutaneous flap with perforators from latissimus dorsi and scapular/parascapular area [13]. Other potential solutions may have been to mobilize the margins of the defect to reduce the overall size. Another option is to consider multiple smaller rhomboid flaps or other local flap such as a keystone flap to reduce tension and recruit tissue around the defect [6].

We acknowledge that the senior author has an extensive experience with rhomboid flaps and felt that in his hands, the final result would likely be most successful with this design. The major limitation of this technique is in patients with lower body mass index and with less available skin [6].

Our results are consistent with other reports in the literature that note the successful applicability of rhomboid flaps in almost all parts of the body. We believe for high safety, high patient satisfaction, and best cosmetic outcomes, rhomboid and other local flaps should be considered as a first-line reconstructive strategy for covering defects of various sizes and locations.

The rhomboid flap design is simple, flap elevation is quick, and no special instrumentation is required, making this technique suitable even in a resource-limited environment.

## 4 What does this case report add?

This report is unique in that it is supported by detailed photographical documentation of each operative stage of the management. The excision design, the location of perforator, the excised specimen, the flap elevation, flap rotation, flap inset, and post-operative photographs are all included to aid readers in utilizing this technique. We urge the readers to consider rhomboid flap as a first-line reconstructive strategy for defect of any size and location.

## 5 Conclusion

Our case report demonstrates the expanding applicability of rhomboid flap with an easy design, quick elevation, safe inset, and successful outcome even in the setting of a very large cutaneous defect. The final flap healed with excellent contour, texture, thickness, and color match.

## Ethical approval

No Institutional Review Board approval needed for case report at our institution.

## Sources of funding

No sponsor and no funding

## Author contribution

AK, senior author, performed the intervention. Both authors, AK and KK, contributed to manuscript. Both authors have read and agreed with the manuscript.

## Registration of research studies

1. Name of the registry: NA. This is a case report of one patient.
2. Unique Identifying number or registration ID:
3. Hyperlink to your specific registration (must be publicly accessible and will be checked):

## Guarantor

Ajaipal s. Kang, MD FACS.

## Consent

Written informed consent was obtained from the patient for publication of this case report and accompanying images. A copy of the written consent is available for review by the Editor-in-Chief of this journal on request.

## Patient/guardian consent

The patient has given consent for possible publication of this case report.

## Provenance and peer review

Not commissioned, externally peer-reviewed.

## Declaration of competing interest

No conflict of interest from both authors.

## References

[1] Gottlieb L.J., Krieger L.M. (1994). From the reconstructive ladder to the reconstructive elevator. Plast. Reconstr. Surg..

[2] Aydin O.E., Tan O., Algan S., Kuduban S.D., Cinal H., Barin E.Z. (2011). Versatile use of rhomboid flaps for closure of skin defects. Eurasian J. Med..

[3] Chasmar L.R. (2007). The versatile rhomboid (Limberg) flap. Can. J. Plast. Surg..

[4] Alvarez G.S., Laitano F.F., Siqueira E.J., Oliveira M.P., Martins P.D.E. (2012). Use of the rhomboid flap for the repair of cutaneous defects. Rev. Bras. Cir. Plást..

[5] Quaba A.A., Sommerlad B.C. (1987). A square peg into a round hole”: a modified rhomboid flap and its clinical application. Br. J. Plast. Surg..

[6] Kang A.S., Kang K.S. (2020). Rhomboid flap for large cutaneous trunk defect. Plast. Reconstr. Surg. Glob. Open.

[7] Agha R.A., Franchi T., Sohrabi C., Matthew for the Scare group G. (2020). The SCARE 2020 guideline: updating consensus surgical CAse REport (SCARE) guidelines. Int. J. Surg..

[8] Janis J.E., Kwon R.K., Attinger C.E. (2011). The new reconstructive ladder: modifications to the traditional model. Plast. Reconstr. Surg..

[9] Borges A.F. (1981). The rhombic flap. Plast. Reconstr. Surg..

[10] Kang A.S., Kang K.S. (2020). A systematic review of cutaneous dog ear deformity: a management algorithm. Plast. Reconstr. Surg. Glob. Open.

[11] Tilleman T.R., Neumann MH M.H., Smeets N.W., Tilleman M.M. (2006). Waste of skin in elliptical excision biopsy of non-melanomatous skin cancer. Scand. J. Plast. ReConstr. Surg. Hand Surg..

[12] Horwood J., Hanratty D., Chandran P., Billings P. (2012). Primary closure or rhomboid excision and Limberg flap for the management of primary sacrococcygeal pilonidal disease? A meta-analysis of randomized controlled trials. Colorectal Dis..

[13] Nassif T.M., Vidal L., Bovet J.L., Baudet J. (1982). The parascapular flap: a new cutaneous microsurgical free flap. Plast. Reconstr. Surg..

